# Herbal Medicine Use During Pregnancy: A Review of the Literature With a Special Focus on Sub-Saharan Africa

**DOI:** 10.3389/fphar.2020.00866

**Published:** 2020-06-09

**Authors:** Magalie El Hajj, Lone Holst

**Affiliations:** ^1^Centre for International Health, University of Bergen, Bergen, Norway; ^2^Medical Affairs, Partner 4 Health, Paris, France; ^3^Department of Global Public Health and Primary Care, University of Bergen, Bergen, Norway; ^4^Centre for Pharmacy, University of Bergen, Bergen, Norway

**Keywords:** herbal medicine, pregnancy, sub-Saharan Africa, traditional medicine, ethnopharmacology

## Abstract

Herbal medicine use has grown considerably worldwide among pregnant women, and is particularly widespread in sub-Saharan Africa. However, herbal medicines used across sub-Saharan Africa are associated with important research gaps and a lack of regulatory framework. This is particularly problematic, as herbal medicine use during pregnancy raises several concerns attributed to the herbal ingredient itself, conventional drug-herbal medicine interactions, and contamination or adulteration of herbal remedies. Moreover, several local herbal remedies used by sub-Saharan African pregnant women have never been botanically identified. In this review, an overview of the practice of herbal medicine, including the regulations, challenges and overall safety, is provided. Then, we discuss the prevalence of herbal medicine use during pregnancy across different sub-Saharan African countries, as well as the indications, adverse outcomes, and effectiveness of the most commonly used herbal medicines during pregnancy in that region.

## Introduction

Pregnancy is characterized by significant physiological changes resulting in various symptoms, such as nausea, vomiting, heartburn, and constipation. These ailments often cause pregnant women to resort to self-medication including the use of herbal medicines (John and Shantakumari, 2015). Consequently, the use of herbal medicines has grown considerably worldwide among pregnant women (Kennedy et al., 2013; Bayisa et al., 2014; John and Shantakumari, 2015). The prevalence of herbal medicine use during pregnancy varies significantly, depending on the geographic location, ethnicity, cultural traditions, and socioeconomic status (Illamola et al., 2020). In a multinational, cross-sectional, internet-based study conducted among 9,459 pregnant women from 23 countries in Western Europe (n = 3,201), Northern Europe (n = 2,820), Eastern Europe (n = 2,342), North America (n = 533), South America (n = 346), and Australia (n = 217), 28.9% of women reported herbal medicine use during pregnancy. Russia (69.0%), Poland (49.8%), and Australia (43.8%) had the highest reported rates of herbal medicine users (Kennedy et al., 2013). The prevalence of herbal medicine use in pregnancy has also been reported in various other countries, including Iran (49.2%), Egypt (27.3%), Bangladesh (70.0%), Iraq (53.7%), Palestine (40.0%), and Taiwan (33.6%) (Chuang et al., 2009; Khadivzadeh and Ghabel, 2012; Al-Ramahi et al., 2013; Orief et al., 2014; Hwang et al., 2016; Ahmed et al., 2018a).

Previous research has shown that herbal medicine use during pregnancy is quite common across sub-Saharan Africa (Ahmed et al., 2018b). However, in several sub-Saharan African countries, there is a paucity of data on the use of herbal medicines among pregnant women. The following paper presents an in-depth literature review which aims to provide the best available information on herbal medicine (safety, regulations, and challenges). This review also identifies the prevalence of herbal medicine use during pregnancy across different sub-Saharan African countries, and summarizes information on the indications, adverse outcomes, and effectiveness of the most commonly used herbal medicines during pregnancy in that region. Herein, we reviewed both published and unpublished studies, drafted in English or French, reporting on herbal medicine use during pregnancy in any sub-Saharan African country, including observational studies, qualitative studies, and meta-analyses. A systematic literature search was conducted between December 2018 and February 2020, by searching the electronic databases MEDLINE and Google Scholar, and through checking reference lists for the identification of additional, relevant studies.

## Challenges Associated with the Herbal Medicine Practice

**Figure 1:**
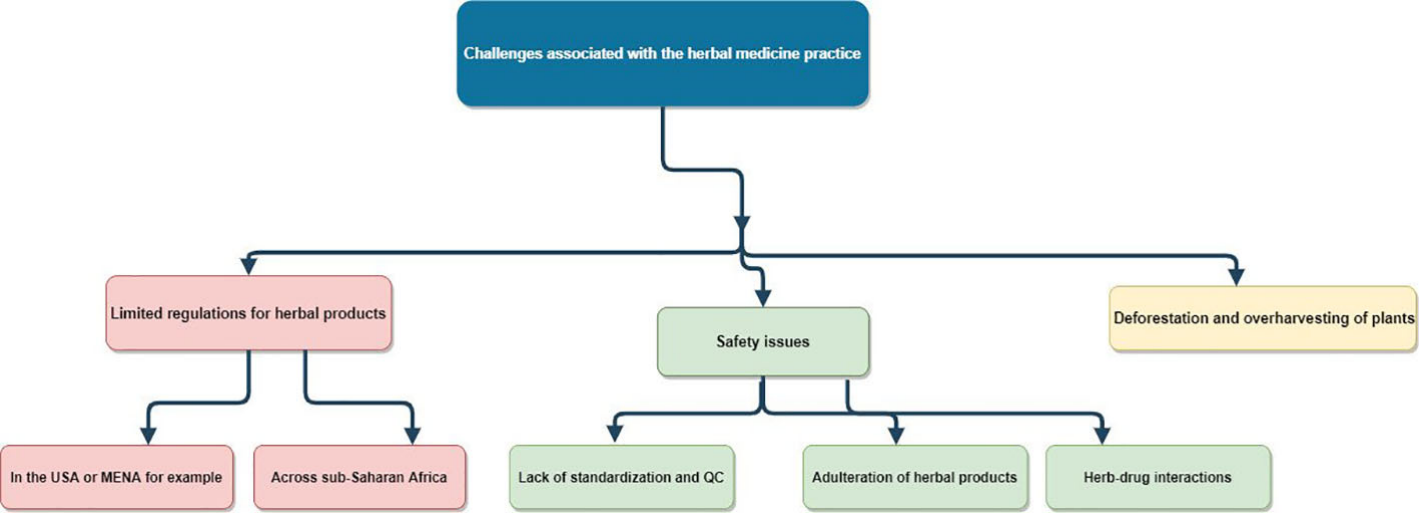
Challenges associated with the herbal medicine practice. MENA, Middle East and North Africa; QC, quality control; USA, United States of America.

According to the World Health Organization, there are several challenges associated with the herbal medicine practice (**Figure 1**), which are related to regulatory status, assessment of safety and efficacy, quality control, safety monitoring, and lack of knowledge about herbal medicine within national drug regulatory authorities (World Health Organization, 2005).

The diversity of herbal remedies and their uses between different countries makes scientifically evaluating and regulating them very challenging (Wachtel-Galor and Benzie, 2011). Hence, in some countries, herbal medicines are subjected to rigorous manufacturing standards, while in others, they are regarded as food supplements for which therapeutic claims are prohibited. For example, in Europe, the Directive 2001/83/EC and the Directive 2004/24/EC divide herbal medicines into two categories: “well-established herbal medicinal products” and “traditional herbal medicinal products”. For the first category, it is needed to demonstrate with sound bibliographic data that the herbal medicinal product has a well-established medicinal use with recognized efficacy and an acceptable level of safety. The herbal medicine product should have a history of safe and efficacious use for at least 10 years within the European Union for a well-established use registration. For the second category, manufacturers of herbal medicines are required to demonstrate that the safety and effectiveness of the traditional herbal medicinal product are based on long-standing medicinal use: 30 years worldwide, including at least 15 years in the European Union (Wachtel-Galor and Benzie, 2011). In the United States, by contrast, the Dietary Supplement Health and Education Act of 1994 classifies herbal products as dietary supplements, a product category that does not require pre-approval. Consequently, the manufacturer is responsible for ensuring that a herbal product is safe before it is marketed, and the Food and Drug Administration can only take regulatory action against any unsafe herbal product after it reaches the market. Therefore, in contrast to prescription and newer over-the-counter medications, herbal products in the United States are usually marketed without the benefit of clinical trials to demonstrate either efficacy or safety (Broussard et al., 2010). In sub-Saharan Africa, the development of national policies and regulations for herbal medicines is substantially more limited, resulting in the proliferation of both locally produced and imported herbal remedies of dubious quality, safety, and efficacy (World Health Organization, 2005). For instance, in Zambia, although the Medicines and Allied Substances Act of 2013 mandates the Zambia Medicines Regulations Authority to regulate and control the sales of herbal medicines, there is neither a system of registration nor a post-marketing surveillance system for herbal medicines (World Health Organization, 2005; Lusaka Times, 2018). Similarly, in Kenya, although the 2005 National Policy on Traditional Medicine and Medicinal Plants stressed the need to take inventory of all herbal medicines available in the country, these recommendations have yet to be implemented (Okumu et al., 2017).

Besides the use of inherently toxic medicinal plants, the toxicity problems of herbal medicines can be attributed to a lack of proper standardization and quality control in the formal herbal industry (Mensah et al., 2019; Ozioma and Chinwe, 2019). Indeed, there is a close correlation between the safety and efficacy of herbal medicines and the quality of the source materials used in their production. The quality of source materials is, in its turn, determined by intrinsic (genetic) and extrinsic (environmental) factors (e.g., exposure to light; availability of water; temperature of processing; period, time, and method of harvest; drying, packing, transportation, and storage of raw herbal material, etc.). Therefore, it is challenging to perform quality controls on the raw materials of herbal medicines, especially given that many countries lack operative machinery needed to implement good manufacturing practices (World Health Organization, 2005). Quality control of herbal medicines is also made more difficult by the seasonal variations in the content of the medicinal plant parts and thus the potential quantitative changes in the composition and/or ratios of bioactive ingredients (Anwar et al., 2016). In addition, there is a paucity of research on whole herbal mixtures partially because the drug approval process does not accommodate undifferentiated mixtures of natural chemicals (Wachtel-Galor and Benzie, 2011). Another source of toxicity of herbal medicines that is important to mention is adulteration of herbal products with undeclared pharmaceutical drugs and potentially toxic compounds such as harmful molecules present in other adulterating botanicals, pathogenic microorganisms, toxins, pesticides, agrochemical residues, and heavy metals (e.g., lead, mercury, and arsenic) (Saper et al., 2004; Mensah et al., 2019). Incorrect identification of plants may also lead to the inadvertent use of potentially toxic species. Furthermore, toxicity may arise as a result of herb-drug interactions (Ozioma and Chinwe, 2019). For example, it has been shown that a pharmacokinetic interaction exists between the antimalarial drug amodiaquine and *Moringa oleifera* Lam., a commonly used medicinal plant with multiple health benefits, when given together or following a long period of ingestion of *Moringa oleifera* Lam. In the presence of *Moringa oleifera* Lam., the peak plasma concentration of amodiaquine decreased by up to 40%, as well as its area under the plasma concentration-time curve which decreased by approximately 11%, leading to a significantly delayed absorption of amodiaquine (Olawoye et al., 2018). Overall, establishing herbal toxicity can be difficult. Even when herbal-related toxicity is suspected, a definitive diagnosis is difficult to establish without proper analysis of the product or plant material (Ozioma and Chinwe, 2019).

Because of the potential toxicity and safety concerns related to herbal products, there is a need to establish a pharmacovigilance system of herbal medicines that is integrated within communities and health facilities, so that data regarding their composition, preparation, indications, and adverse events can be gathered (Ahmed et al., 2018a). The latency period between the use of an herbal product and the occurrence of an adverse event should also be determined, if possible, as this can help in causality assessment in pharmacovigilance management. Such information can facilitate decisions on further protective measures to be taken concerning the future use of herbal medicines (Ozioma and Chinwe, 2019). Research is also needed to meet the challenges of identifying the active compounds in the plants, and there should be research-based evidence on the safety and efficacy of both whole herbs and extracted compounds (Wachtel-Galor and Benzie, 2011).

Regrettably, the expanding herbal product market could lead to overharvesting of plants and threaten the rich biodiversity in sub-Saharan Africa. In addition, poorly managed collection and cultivation practices could contribute to the extinction of endangered plant species and the destruction of natural resources (Wachtel-Galor and Benzie, 2011). It has been suggested that 15,000 medicinal plant species are threatened with extinction (Brower, 2008). Another negative consequence of this trend is that there will be essentially less choice for the future development of medicines, if the situation is not addressed (Brower, 2008). Under these circumstances, forest laws are of considerable importance, as they have a role in ensuring the sustainable availability of medicinal plants. A number of countries in sub-Saharan Africa have enacted forest laws, including the Lesotho Forestry Act of 1978, the Tanzania Forest Act of 2002, the Namibia Forest Act No. 12 of 2001, the Mozambique Forest and Wildlife Act of 1999, the South Africa National Forests Act No. 84 of 1998, the Zambia Forests Act of 1999, and the Zimbabwe Forest Amendment Act of 1999 (Moshi and Mhame, 2013). However, like other existing laws, these forest laws have deficiencies, such as failure to link the forest sector development with economic and social development objectives, weak forest administrative structures, low or poor compensation being given to the local communities that are custodians of the forest resources, poor definition of legal and institutional frameworks regarding forest management and use, and land tenure problems (Moshi and Mhame, 2013). Hence, more international efforts are needed for the preservation of plant populations.

## Overall Safety of Herbal Medicine During Pregnancy

The indications for the use of herbal medicines during pregnancy may vary across regions and countries, and can be mother- or child-related (Illamola et al., 2020). Herbal medicines may be used sometimes as part of maternal care to treat pregnancy-related problems, and often to improve the well-being of the mother and/or the unborn child. The most commonly reported indications are nausea and vomiting, urinary tract infections, preparation for and/or facilitation of labor, common cold or flu, gastrointestinal problems (e.g., constipation, flatulence), pain conditions, improvement of fetal outcomes, prevention of miscarriage, relief of anxiety, treatment and/or prevention of anemia, and treatment of edema (Kennedy et al., 2013; John and Shantakumari, 2015; Ahmed et al., 2018b; Illamola et al., 2020).

As previously mentioned, safety concerns related to herbal products can be mainly attributed to the herbal ingredient itself, conventional drug-herbal medicine interactions, and contamination or adulteration of herbal remedies with potentially toxic compounds such as heavy metals (Broussard et al., 2010). However, herbal medicine use during pregnancy raises particular concerns, because many herbal products are specifically marketed for symptoms that occur commonly during pregnancy, such as nausea and vomiting (Allaire et al., 2000; Broussard et al., 2010). More importantly, there is a lack of randomized controlled trial data on the efficacy and safety of herbal medicine use during pregnancy. In many areas of sub-Saharan African, secrecy surrounds the practice of herbal medicine, with the components and preparation methods of herbal medicines, especially those intended for the treatment of serious ailments such as malaria and HIV/AIDS, exclusively residing with traditional healers (International Bioethics Committee, 2013; Ozioma and Chinwe, 2019). The fact that there are several plants used in African traditional medicine, for which little information is available on their constituents, can increase the risk of adverse reactions, particularly in vulnerable groups such as older adults, children, and pregnant women (Mensah et al., 2019; Ozioma and Chinwe, 2019). The use of unstudied herbal agents with unknown pharmacologic activity can pose a potential risk to both the pregnant women and their fetuses (Allaire et al., 2000; Ahmed et al., 2018a). In a 2004 study investigating herbal medicine use during pregnancy among 400 Norwegian women, approximately 40% of the 144 herbal medicine users consumed herbal products that were either potentially harmful or with missing information about their safety in pregnancy (Nordeng and Havnen, 2004). Similarly, in a large, multinational study which classified 126 different herbal medicines used in pregnancy according to their safety, only 28 herbs (22.2%) were deemed as safe to use in pregnancy based on current literature (Kennedy et al., 2016). More recently, a systematic review, which aimed to determine whether herbal medicinal use during pregnancy was associated with adverse maternal or child outcomes, found that topical use of almond oil was significantly associated with preterm birth, oral raspberry leaf with caesarean delivery, and heavy liquorice consumption (greater than 500 mg/week) with early preterm birth (Munoz Balbontin et al., 2019). In addition, the use of herbal medicines in pregnancy constitutes a major challenge for health care providers, since pregnant women often consume herbal medicines without consulting them (Illamola et al., 2020). Hence, in order to further advance research on the benefits and adverse effects of herbal medicine use during pregnancy, it is important to determine the extent of herbal medicine use during pregnancy in different settings and to find out which herbs pregnant women use.

## Prevalence of Herbal Medicine Use During Pregnancy in Sub-Saharan Africa

**Figure 2:**
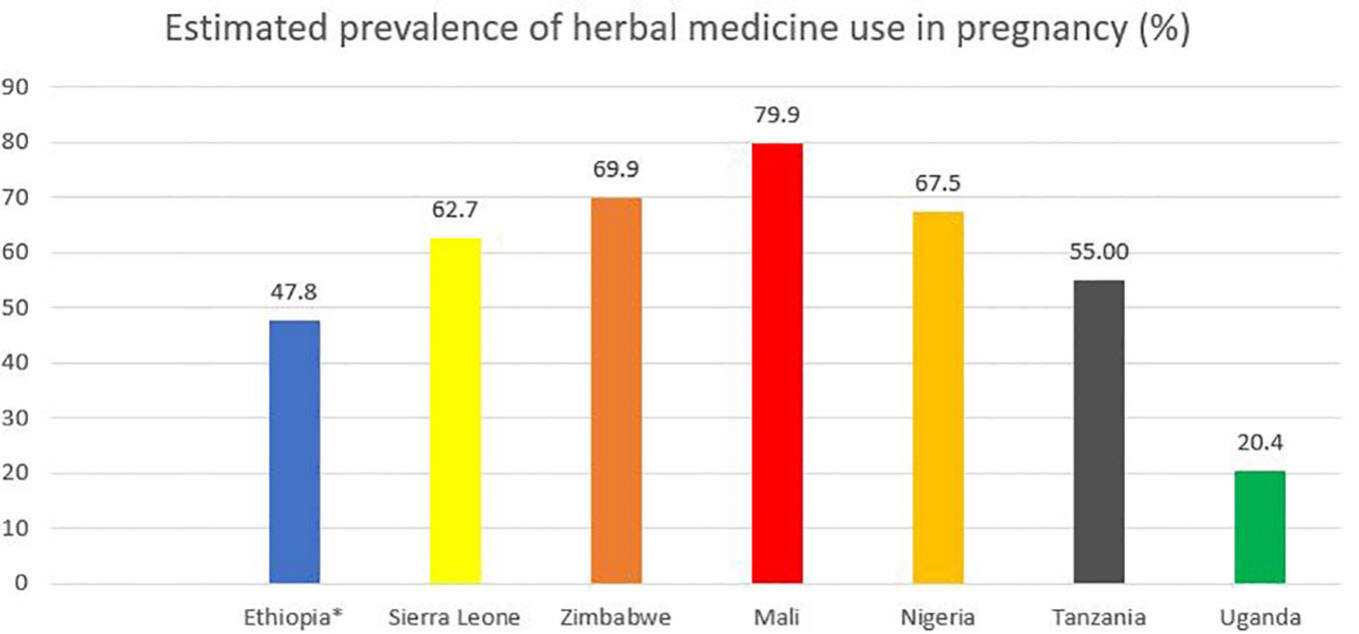
Estimated prevalence of herbal medicine use during in pregnancy in selected sub-Saharan African countries. *The prevalence rate for Ethiopia was based on a recent meta-analysis of eight studies (Adane et al., 2020).

Sub-Saharan Africa is one region of the world in which herbal medicine is traditionally and culturally acknowledged (James et al., 2018b). However, evidence from sub-Saharan Africa suggests wide variability in the use of herbal medicines during pregnancy (**Figure 2**), from 2.0% as reported in a study conducted in the Tigray Region, Northern Ethiopia (Gebremedhin and Gomathi, 2014) to 100% according to another study in Machakos District, Eastern Kenya (Kaingu et al., 2011). It is difficult to ascertain whether these differences in prevalence across studies are caused by variability in study design, setting, data collection, and sampling techniques or whether they represent true differences in herbal medicine use (Kennedy et al., 2013; James et al., 2018a). In a recently published systematic review by Ahmed et al. (2018b) of 50 studies, including a total of 22,404 African pregnant or lactating women, the average prevalence of herbal medicine use during pregnancy among the different African regions was between 32% (in Central Africa) to 45% (in East Africa). This systematic review also found that herbal medicine use during pregnancy was statistically significantly associated (p < 0.05) with a lower educational level, increasing age, being married, low socioeconomic status, lower educational level of the spouse, poor pregnancy outcomes, herbal medicine use in prior pregnancies, perception that medicinal plants are effective, large family size, self-employment, unemployment, and rural residence (Ahmed et al., 2018b). **Table 1** illustrates the characteristics and findings of selected cross-sectional studies evaluating the use of herbal medicines among pregnant women in different sub-Saharan countries.

**Table 1:** Characteristics of cross-sectional studies evaluating herbal medicine use during pregnancy in sub-Saharan Africa.

Reference	Study setting	Sample size	Prevalence of use	Types of medicinal plants used	Indications	Characteristics of users	Disclosure of herbal use to health care providers
(Banda et al., 2007)	Lusaka, Zambia	1,128	21%	Not reported	Not reported	- Users were not different from non-users in terms of age, education, ethnicity, or income - Users were more likely to drink alcohol during pregnancy, have at least two sex partners, engage in “dry sex”, initiate sex with their partner, report a previously treated sexually transmitted disease, and use contraception (all p < 0.01)	64% of users did not want to share their use of herbal medicine to health care providers
(Maluma et al., 2017)	Lusaka Province, Zambia	273	32%	Indigenous local plants: “*Moono”*, *“Makole”*, “*Mulolo”, “Sope”*	Inducing or accelerating labor	- Herbal medicine use was not associated with residence area, age, or education level - Sociocultural beliefs were the major factors that contributed to use of herbal medicine during pregnancy - Most users were unaware of health risks associated with administering crude herbal extracts during different trimesters of pregnancy	Not reported
(James et al., 2018a)	Freetown, Sierra Leone	134	62.7%	*Luffa acutangula* (L.) Roxb., lime leaves (*Citrus aurantiifolia* (Christm.) Swingle), ginger	Urinary tract infections, pedal edema, to improve fetal outcomes	- Pregnant women who identified as Muslims were 3.4 times more likely (p = 0.006) than Christian women to use herbal medicine - Perceived effectiveness and safety of herbal medicines over conventional medicines (70.2%) was the main reason for use	95.2% of users did not disclose their herbal medicine use to their conventional health care providers
(Mureyi et al., 2012)	Harare, Zimbabwe	248	52%	*Pouzolzia mixta* Sohms, cocktails of unknown herbs, okra (*Abelmoschus esculentus* (L.) Moench)	For widening of birth canal, labor induction, nutritional supplement	- Herbal medicine use in pregnancy was significantly associated with being in the 20–25 age group (p = 0.021), nulliparity (p = 0.004), nulligravidity (p = 0.002), and residing in a high-density neighborhood (p = 0.04) - Almost all herbal medicine interventions were employed beginning at onset of the third trimester	Not reported
(Mawoza et al., 2019)	Rural Zimbabwe	398	69.9%	*Fadogia ancylantha* Schweinf., okra (*Abelmoschus esculentus* (L.) Moench), chir pine (*Pinus roxburghii* Sarg.)	To facilitate childbirth, for widening of birth canal	No association was noted between herbal medicine use and any sociodemographic characteristic	Not reported
(Godlove, 2011)	Mbeya, southwest Tanzania	400	55%	Not reported	Labor induction, to improve fetal outcomes	- The use of herbal medicines during pregnancy was associated with long distance to the nearest public health facility, and low education level (all p ≤ 0.01) - The insufficient effectiveness of conventional medicines (64.1%) and the accessibility of herbal medicines (30.5%) were reported as the main reasons for use	Not reported
(Bayisa et al., 2014)	Nekemte, Western Ethiopia	250	50.4%	Ginger, garlic, Tena Adam (*Ruta chalepensis* L.), eucalyptus (*Eucalyptus globulus* Labill.)	For treatment of nausea, morning sickness, vomiting, cough	- Age, educational status, marriage, ethnicity, and source of information were not associated with herbal medicine use - About 70% of users were pregnant women on their first trimester	Not reported
(Laelago et al., 2016)	Hossana, Southern Ethiopia	363	73.1%	Garlic, ginger, Tena Adam (*Ruta chalepensis* L.), Dama Kesse (*Ocimum lamiifolium* Hochst. ex Benth.), eucalyptus (*Eucalyptus globulus* Labill.)	Management of nausea, vomiting, abdominal pain, common cold	Being in the first trimester of pregnancy, having less education, and having less knowledge about herbal medicine favored the use of medicinal plants	Not reported
(Mekuria et al., 2017)	Gondar, Northern Ethiopia	364	48.6%	Ginger, Dama Kesse (*Ocimum lamiifolium* Hochst. ex Benth.)	Common cold, inflammation	- Rural residency, having no formal education, and having an average monthly income <100 United States Dollars were found to be strong predictors of herbal medicine use - 68.4% of users consumed herbal medicines during their third trimester	89.8% of users had not consulted their doctors about their herbal medicine use
(Fakeye et al., 2009)	North Central, North West and South West, Nigeria	595	67.5%	Not reported	Not reported	- Age (p = 0.003), geographical zones (p = 0.02), and educational status (p = 0.04) were significantly associated with herbal medicine use - Users used medicinal plants because they perceived them as being more effective than conventional medicines (22.4%), and safe (21.1%) - 56.6% of participants did not support combining herbal medicines with conventional medications to forestall drug-herb interaction	Not reported
(Tamuno et al., 2010)	Kano, North West Nigeria	500	31.4%	Ginger, garlic	Not reported	- Use of herbal medicine was significantly associated with no formal education and low socioeconomic status (p < 0.05 for both) - Over 40% of women reported combined use of herbs and drugs	Not reported
(Duru et al., 2016)	Owerri, South East Nigeria	500	36.8%	Bitter leaf (*Gymnanthemum amygdalinum* (Delile) Sch.Bip.), palm kernel oil, bitter kola (*Garcinia kola* Heckel)	Not reported	Being married (p < 0.001), having no formal education (p < 0.001), and having a monthly income >250 USD (p = 0.003) were significantly associated with herbal medicine use during pregnancy	Not reported
(Nergard et al., 2015)	One urban and two rural regions, Mali	209	79.9%	*Lippia chevalieri* Moldenke, *Combretum micranthum* G. Don, *Parkia biglobosa* (Jacq.) R.Br. ex G.Don, *Vepris heterophylla* (Engl.) Letouzey	For general well-being, to treat malaria symptoms, edema, urinary tract infection, tiredness	- Sociodemographic characteristics were not associated with the use of herbal medicines - Frequent use of herbal medicines was reported during the first semester	Pregnant women used herbal preparations without any supervision from their health care providers
(Mothupi, 2014)	Nairobi, Kenya	333	12%	Not reported	To treat toothache, back pain, flu, indigestion, swollen feet	- The use of herbal medicine was associated with a lower level of education (p = 0.007), and use before the index pregnancy (p < 0.001) - 51% of users reported use of combined herbs with pharmaceutical drugs	Only 12.5% of users disclosed the use of herbal medicines to health care professionals
(Nyeko et al., 2016)	Gulu District, Northern Uganda	383	20.4%	Local herbs (not reported)	To treat abdominal/waist pain, fever, skin problems, nausea and vomiting, and for induction of labor	- Women who used herbal medicines in their previous pregnancies were 8 times more likely to use them during the current pregnancy - Residing more than 5 km from the nearest health facility was associated with increased herbal medicine	89.7% of the users of herbal medicines did not disclose the use of local herbs to their health care providers
(Adusi-Poku et al., 2016)	Offinso North District, Ghana	384	6.5%	*Senna occidentalis* (L.) Link, *Sida acuta* Burm.f., *Cola gigantea* A.Chev.	To ease labor and to improve fetal outcomes	High usage was found among married women, and among those with no formal education, and women with median age of 25 years	Not reported

The popularity of herbal medicines among pregnant women can be mainly attributed to the belief that herbal products, being natural, are safe with fewer adverse events compared to conventional drugs (Ernst, 2002; Barnes et al., 2018; Peprah et al., 2019). Additionally, several studies have shown that pregnant women in sub-Saharan Africa, particularly in rural areas, use herbal medicines because they consider them less costly and more accessible than conventional drugs (Bayisa et al., 2014; Batisai, 2016; Ahmed et al., 2018a; Peprah et al., 2019). Of note, the ratio of traditional healers to the population in sub-Saharan Africa is 1:500, whereas the ratio of medical doctors to the population is 1:40,000, and most medical doctors are concentrated in urban areas and cities at the expense of rural areas (Abdullahi, 2011). Herbal medicine use was also shown to be in line with the sociocultural, religious and spiritual values of the people who use it in sub-Saharan Africa (Batisai, 2016; James et al., 2018b; Gakuya et al., 2020). Indeed, in many parts of sub-Saharan Africa, traditional medicine defines illness as a consequence of a breakdown of social balance (e.g., breaking codes of conduct in the present or in the past, ancestor or evil spirits, as well as witchcraft or fate) (Ahlberg, 2017; Stephani et al., 2018). Moreover, according to Pretorius (1991), the use of herbal medicine post-independence became one of the ways sub-Saharan Africans deployed to “rediscover their sociocultural identity”, and to deal with inaccessible and expensive medicines foreign to them (Pretorius, 1991).

## Most Commonly Used Herbal Medicines During Pregnancy in Sub-Saharan Africa

Due to differences in culture, traditions, and climate, it is expected that herbal medicines used during pregnancy vary across countries and regions. In the multinational study by Kennedy et al. (2013) in 9,459 pregnant women from 23 countries in Europe, North and South America, and Australia, the most frequently used herbal medicines were *Zingiber officinale* Roscoe (ginger), *Vaccinium oxycoccus* L./*Vaccinium macrocarpon* Aiton L. (cranberry), *Valeriana officinalis* L. (valerian), *Rubus idaeus* L. (raspberry leaf), *Matricaria chamomilla* L. (chamomile), and *Mentha piperita* L. (peppermint) (Kennedy et al., 2013). The African continent, generally known for its rich biodiversity, has an estimated total flora of over 70,000 species (Govaerts, 2001). This species richness was reflected in the systematic review by Ahmed et al. in which a total of 274 different medicinal plant species from 87 plant families were reported to be used during pregnancy (Ahmed et al., 2018b). The most commonly cited medicinal plant species were *Zingiber officinale* Roscoe (ginger), *Allium sativum* L. (garlic), *Cucurbita pepo* L. (pumpkin), *Gymnanthemum amygdalinum* (Delile) Sch.Bip. (syn. *Vernonia amygdalina* Delile) (bitter leaf), *Ricinus communis* L. (castor oil), *Garcinia kola* Heckel (bitter kola), *Ocimum lamiifolium* Hochst. ex Benth. (basil or Dama Kesse in Amharic), *Azadirachta indica* A. Juss (neem), *Ruta chalepensis* L. (Tena Adam, in Amharic; fringed rue in English), and *Aloe vera* (L.) Burm.f. (*Aloe vera*) (Ahmed et al., 2018b). **Table 2** presents the most commonly used herbal medicines during pregnancy, based on the Kennedy et al. (2013) multinational study and the Ahmed et al. (2018b) systematic review.

**Table 2:** Most commonly used herbal medicines during pregnancy: indications, reported adverse events, and preparations.

Common name(s)	Binomial name(s)	Indication(s)	Reported adverse events	Common preparation(s)	References
Ginger	*Zingiber officinale* Roscoe	Nausea, vomiting	Drowsiness, reflux, vomiting, heartburn, headache, abdominal discomfort, preterm delivery, smaller head circumference of newborns	Ginger tea; chewing raw ginger	(Holst et al., 2011; Dante et al., 2014; Trabace et al., 2015)
Cranberry	- *Vaccinium oxycoccos* L. - *Vaccinium macrocarpon* Aiton	Urinary tract infections	Gastrointestinal upset, spotting in the second and third trimesters	Cranberry juice	(Holst et al., 2011; Heitmann et al., 2013; Dante et al., 2014)
Valerian	*Valeriana officinalis* L.	Sleep disorders	Diarrhea	Root decoction; capsules	(Bent et al., 2006)
Raspberry leaf	*Rubus idaeus* L.	Nausea, increase in milk production, labor induction	Hypoglycemia, higher percentage of cesarean deliveries versus non-users	Raspberry leaf tea; capsules	(Nordeng et al., 2011; Al-Ramahi et al., 2013; Cheang et al., 2016)
Chamomile	- *Matricaria chamomilla* L. - *Chamaemelum nobile* (L.) All.	Gastrointestinal irritation, insomnia, joint pain, relaxation	Breast engorgement and tenderness, low birth weight, preterm delivery	Chamomile tea prepared from dried flowers	(Al-Ramahi et al., 2013; Trabace et al., 2015; Silva et al., 2018)
Peppermint	*Mentha piperita* L.	Nausea, vomiting, flatulence, indigestion, irritable bowel syndrome	Heartburn, dry mouth, belching, rash, dizziness, headache	Leaf tea; oil extract	(Al-Ramahi et al., 2013; Alammar et al., 2019)
Garlic	*Allium sativum* L.	Prophylaxis of preeclampsia, preterm birth prophylaxis, enhancing the immune system	Foul odor, nausea	Eating raw garlic; garlic tea; garlic juice	(Ziaei et al., 2001; Myhre et al., 2013; Laelago, 2018)
Pumpkin	*Cucurbita pepo* L.	Nutritional supplement, cough, fever, common cold, headache, heartburn, gastrointestinal irritation, edema	No adverse events were identified in the literature	Decoction of seeds; leaf juice (mixed with milk)	(Adnan et al., 2017; Ahmed et al., 2018b)
Bitter leaf	*Gymnanthemum amygdalinum* (Delile) Sch.Bip.	Nausea, vomiting, fever, constipation, increasing appetite, strengthening the pelvic floor muscles, malaria, anemia	Stimulation of uterine motility	Leaf soup; fresh leaves are washed and squeezed in clean water and the water is extracted for drinking	(Attah et al., 2012; Olowokere and Olajide, 2013; Nalumansi et al., 2017)
- Castor oil - Castor bean	*Ricinus communis* L.	Inducing labor	Nausea, abdominal pain, uterine rupture	Leaves or roots soaked in hot or cold water, usually drank at labor onset	(Sicuranza and Figueroa, 2003; Dante et al., 2014; Gilad et al., 2018)
Bitter kola	*Garcinia kola* Heckel	Nausea, vomiting	Weight loss, prolonged sleep duration, increased libido	Chewing the seeds	(Adegbehingbe et al., 2008; Ahmed et al., 2018b)
- Dama Kesse (in Amharic) - Basil	*Ocimum lamiifolium* Hochst. ex Benth.	Headache, fever, inflammation, joint pain, back pain, common cold, cough, eye infections	No adverse events were identified in the literature	Oral decoction of crushed leaves; leaf juice drank or sniffed or used as an eye drop	(Hailemeskel et al., 2017; Nega et al., 2019)
- Neem - Nimtree - Indian lilac	*Azadirachta indica* A. Juss	Inducing labor, malaria, pain, hemorrhoids, enhancing fetal development	Vomiting, metabolic acidosis, encephalopathy	Body smeared with mashed leaves; neem leaf tea; oral neem bark extracts	(Mishra and Dave, 2013; Malan et al., 2015; Dika et al., 2017; Ahmed et al., 2018b)
- Tena Adam (in Amharic) - Fringed rue	*Ruta chalepensis* L.	Nausea, vomiting, common cold, abdominal discomfort	Sedation, drowsiness	Leaf tea; leaf juice	(Al-Said et al., 1990; Ahmed et al., 2018b)
*Aloe vera*	*Aloe vera* (L.) Burm.f.	Digestive problems, constipation, skin treatment	Itching, rash	Topical gel; lotion; leaf tea; leaf juice	(Cuzzolin et al., 2010; Facchinetti et al., 2012; Ahmed et al., 2018b)

Several cross-sectional studies have shown that ginger is commonly used by pregnant women across sub-Saharan Africa for nausea and vomiting during pregnancy (Laelago et al., 2016; Shewamene et al., 2017; Ahmed et al., 2018b). A systematic review of 12 randomized controlled trials involving 1,278 pregnant women found that ginger significantly improved the symptoms of nausea when compared to placebo (p < 0.001), with subgroup analyses favoring the lower daily dosage of <1.5 g of ginger for nausea relief (Viljoen et al., 2014). This finding is not surprising, given that animal studies have shown that ginger can increase gastric contractility, speed up gastric emptying, and consequently increase the gastrointestinal transit time of meals, which can decrease the feeling of nausea (Chrubasik et al., 2005). However, in the systemic review by Viljoen and colleagues, ginger did not have a significant impact on vomiting episodes (p = 0.06 versus placebo). It was also associated with a favorable safety profile, as it did not pose a risk for spontaneous abortions or for other adverse events such as heartburn or drowsiness (Viljoen et al., 2014).

Garlic is characterized by diverse therapeutic properties, including anti-hypertensive, anti- inflammatory, anti-oxidant, anti-bacterial, hypocholesteremic, and anti-cancer properties (Al Disi et al., 2016). In a randomized, single-blind, placebo-controlled study among 100 nulliparous pregnant women, the administration of 800 mg/day of garlic tablets during the third trimester of pregnancy was found to be effective in reducing the occurrence of hypertension (p = 0.043 versus placebo) and in lowering serum cholesterol levels (p = 0.038). However, compared to placebo, garlic consumption was associated with a slightly higher incidence of nausea (Ziaei et al., 2001). A meta-analysis of 18 randomized, placebo-controlled trials also found that garlic supplements induce a significant reduction in both systolic and diastolic blood pressure by 3.75 and 3.39 mmHg, respectively (p < 0.001 compared to placebo) (Wang et al., 2015). Although the mechanism of garlic’s anti-hypertensive effect remains unclear, it has been reported that garlic may elicit its anti-hypertensive effects by inhibition of angiotensin-converting enzymes through its bioactive compound allicin (Wang et al., 2015; Al Disi et al., 2016). It is recommended for pregnant women to avoid using garlic prior to surgery including caesarean section, as garlic was reported to have an anti-hemostatic effect and may consequently interfere with blood clotting (Al Disi et al., 2016; Laelago, 2018).

Similarly to garlic, pumpkin is also known for its multiple beneficial effects, such as anti-diabetic, anti-hypertensive, anti-tumor, anti-bacterial, anti-viral, anti-fungal, hypocholesteremic, anti-ulcer, anti-inflammatory, and analgesic effects (Jafarian et al., 2012; Adnan et al., 2017). Protein-bound polysaccharides and pectines, found in different species of *Cucurbita*, could exert anti-oxidant effects by elevating glutathione peroxidase and superoxide dismutase activity as well as reducing serum levels of malondialdehyde (Mahmoodpoor et al., 2018). In addition, enhancement of splenic lymphocyte proliferation, natural killer cell activity, and an increase in the number of CD4^+^, CD8^+^ T cells, and the CD4^+^/CD8^+^ ratio by pumpkin extracts have been reported, confirming the immuno-modulatory activity of pumpkin (Xia et al., 2003). Despite the long-standing medicinal use of pumpkin, there are no clinical trial data available to support its use during pregnancy and lactation.

Bitter leaf is a perennial plant growing predominantly in tropical Africa. It has gained wide application in Nigeria, Uganda, Ethiopia, Tanzania, and other sub-Saharan African countries for the treatment of malaria, amoebiasis, measles, and helminthic infections, and its use is quite popular among pregnant women (Olowokere and Olajide, 2013; Nalumansi et al., 2017; Oyeyemi et al., 2018). In a single-arm trial from Western Uganda examining the efficacy and safety of an oral bitter leaf infusion in patients aged 12 years and over with uncomplicated malaria, bitter leaf was associated with an adequate clinical response after 2 weeks of treatment in 67% of cases, as well as reduced parasitemia by 32%. No significant adverse events were reported throughout the trial (Challand and Willcox, 2009). Although more than 30 bioactive compounds have been isolated, the sesquiterpene lactones found in the leaves of *Gymnanthemum amygdalinum* have been shown to be the main active compounds responsible for most of the plant’s therapeutic activities (Luo et al., 2011; Abay et al., 2015; Oyeyemi et al., 2018). Despite the absence of experimental studies in pregnant women, a 3-week experiment among 20 pregnant rats found that the aqueous extract of bitter leaf has an abortifacient effect, and is associated with reduced serum progesterone levels. The same study also found a median lethal dose above 5,000 mg/kg, prompting the authors to conclude that bitter leaf is safe for human consumption (Isa et al., 2014). Nevertheless, caution is advised with the clinical use of bitter leaf during pregnancy, and particularly during the first trimester, as it may stimulate uterine contractions (Olowokere and Olajide, 2013).

There is a wide use of castor oil to induce labor. In a recent systematic review and meta-analysis of five randomized controlled trials and five observational studies aimed at comparing pregnancy outcomes between users and non-users of castor oil for labor induction, castor oil users were significantly more likely to give birth within 24 h than non-users (relative risk, 3.46; 95% confidence interval, 1.58–7.55), highlighting the oxytocic properties of this medicinal plant (Zamawe et al., 2018). Castor oil is thought to induce labor *via* its main ingredient ricinoleic acid, a hydroxylated fatty acid released from castor oil by intestinal lipases. Ricinoleic acid specifically activates the prostaglandin EP_3_ receptor resulting in laxation and uterus contraction (Tunaru et al., 2012). With respect to safety, the systematic review by Zamawe and colleagues found no statistically significant differences in the rate of hemorrhage, caesarean section, assisted vaginal delivery, referral to neonatal intensive care unit, meconium-stained liquor, maternal death, stillborn, and uterine rupture between users and non-users of castor oil (Zamawe et al., 2018). Nevertheless, caution should be exercised, as cases of uterine rupture associated with castor oil ingestion have been reported in the literature (Sicuranza and Figueroa, 2003; Fruscalzo et al., 2012).

Bitter kola is a flowering plant found mostly in the tropical rain forest region of Central and West Africa. Although bitter kola is mostly used among pregnant women to treat nausea and vomiting (Ahmed et al., 2018b; Laelago, 2018), reported pharmacological effects of this plant include anti-diabetic, anti-inflammatory, antipyretic, immunomodulatory, anti-atherogenic, anti-microbial, and hepaprotective effects (Okoye et al., 2014). To our knowledge, there are no available clinical trial data on bitter kola in pregnant women. However, a randomized, placebo-controlled, double-blind study of bitter kola in patients with knee osteoarthritis reported a favorable safety profile of bitter kola, with only minor adverse events (i.e., weight loss, prolonged sleep duration, and increased libido) (Adegbehingbe et al., 2008). These adverse events are not surprising, given that bitter kola seeds contain approximately 3% of caffeine and 2% of theobromine. Given their relatively high caffeine content, pregnant women should limit their use.

Dama Kesse is a versatile aromatic genus that is particularly popular in Ethiopia, with anti-malarial, antipyretic, anti-oxidant, anti-spasmodic, and anti-bacterial properties (Kefe et al., 2016; Ahmed et al., 2018b; Sahalie et al., 2018). Despite its widespread use among pregnant women across Ethiopia (Adane et al., 2020), Dama Kesse is very poorly studied, with no clinical studies identified.

Neem is a fast-growing tree that is a member of the Meliaceae family. It is characterized by a broad spectrum of activity including anti-inflammatory, antipyretic, analgesic, cardiovascular, hypoglycemic, diuretic, immunomodulatory, dermatological, anti-bacterial, anti-fungal, anti-ulcer, anti-tumor, and anti-malarial effects. Although its exact mechanism in the prevention of pathogenesis is not entirely understood, the therapeutic activity of neem is thought to result from the synergistic/additive effects of its various bioactive compounds such as azadirachtin, nimbolinin, nimbin, nimbidin, nimbidol, sodium nimbinate, gedunin, salannin, and quercetin (Govindachari et al., 1998; Alzohairy, 2016). Despite being a native to the Indian subcontinent, neem is commonly used across sub-Saharan Africa, particularly for malaria prevention and treatment (Luong et al., 2012; Kumar and Navaratnam, 2013; Malan et al., 2015). Although neem has been extensively investigated in the non-clinical setting, only a handful of clinical studies have been published, of which none were conducted in pregnant women (Alzohairy, 2016). In a small, single-arm clinical trial among 26 adult patients suffering from gastric acidity and gastroduodenal ulcers, the twice daily administration of 30-mg capsules of neem bark extract for 10 days was associated with a significant decrease of gastric acid secretion (by 77% compared to baseline; p < 0.002). The volume of gastric secretion and pepsin activity were also inhibited by 63% and 50%, respectively. Neem showed a favorable safety profile, with no major adverse events reported (Bandyopadhyay et al., 2004). In another small, 6-week trial among 36 patients with oral infections, neem extract dental gel significantly reduced the plaque index and bacterial count compared to those receiving chlorhexidine gluconate (0.2% w/v) mouthwash (Pai et al., 2004). Despite these positive results, case reports in both adults and children have reported neem oil poisoning incidents causing vomiting, seizures, hepatic toxicity, metabolic acidosis, and toxic encephalopathy (Sinniah and Baskaran, 1981; Sundaravalli et al., 1982; Mishra and Dave, 2013). In addition, oral use of purified neem seed extract has been found to be efficacious in pregnancy termination in both rodents and primates, as indicated by a total decline of chorionic gonadotropin and progesterone and by resumption of cyclic changes in hormone profiles and perineal sex swelling in baboons (Talwar et al., 1997). This abortive effect of oral neem seed extracts seems to be propelled by activation of cell-mediated immune reactions (Atawodi and Atawodi, 2009). Hence, despite the absence of reliable clinical trial data, the use of neem preparations during pregnancy should be discouraged.

Tena Adam is a shrubby plant that is cultivated in the highlands of Ethiopia. Similarly to Dama Kesse, it is poorly studied with no clinical trial data available. However, the administration of an aqueous infusion of Tena Adam during organogenic period in pregnant mice led to increased flow and decreased vascular resistance in the placenta bed, as well as fetal thymic involution (Zeichen de Sa et al., 2000). Thus, Tena Adam should also be discouraged in pregnant women until well-designed clinical trials are available.

*Aloe vera* is perhaps one of the most widely used herbal remedies for topical skin conditions. *Aloe vera* extracts improve skin moisture by a humectant mechanism, and prevent skin ulcers as they contain mucopolysaccharides, amino acids, zinc, and water (Dal’Belo et al., 2006; Hekmatpou et al., 2019). Although the topical application of *Aloe vera* is associated with infrequent adverse events (Hekmatpou et al., 2019), orally ingested *Aloe vera* whole leaf extract has shown clear evidence of carcinogenic activity in rats, and was classified by the International Agency for Research on Cancer as a possible human carcinogen (Guo and Mei, 2016). In addition, pregnant women are generally not advised to take oral preparations of *Aloe vera* because its cathartic property might result in stimulating uterine contractions, thereby increasing the risk for premature labor or miscarriage (Guo and Mei, 2016).

## Conclusion

Sub-Saharan Africa is home to an extensive and diverse medicinal plant life. Consequently, the use of herbal medicines is widespread in that region. Despite this, the study of herbal medicine use during pregnancy which is related to maternal health, a public health priority in many sub-Saharan African countries, remains limited (Wekesah et al., 2016). Currently, there is not enough information to recommend the safe use of herbal medicinal products during pregnancy, as most herbal remedies used across sub-Saharan Africa are not backed by robust scientific studies. Moreover, several local herbal remedies used by sub-Saharan African pregnant women have never been botanically identified. This might be due to the lack of detailed documentation of traditional knowledge, which is generally transferred orally. Thus, further research on herbal medicine use during pregnancy is highly warranted in sub-Saharan Africa, as it might prevent maternal mortality and morbidity and decrease the possibility of endangering the health of fetuses.

A low disclosure rate of herbal medicine use to health care providers has also been identified in the present review. This suggests that collaborative communication by health care providers is important, as it may mitigate against the potential dangers of herbal medicine use during pregnancy and of uncoordinated concurrent herbal and conventional medicine use. Finally, given that traditional and sociocultural influence may play a role in herbal medicine use during pregnancy, there is a need to better understand the reasons and facilitators of herbal medicine use in pregnant women from sub-Saharan Africa.

## Author Contributions

MH drafted the manuscript. LH revised the manuscript.

## Funding

This work was funded by the Centre for International Health, University of Bergen, Norway.

## Conflict of Interest

MH is a Partner 4 Health employee.

The remaining author declares that the research was conducted in the absence of any commercial or financial relationships that could be construed as a potential conflict of interest.
